# Predictors of Uric Acid Stones: Mean Stone Density, Stone Heterogeneity Index, and Variation Coefficient of Stone Density by Single-Energy Non-Contrast Computed Tomography and Urinary pH

**DOI:** 10.3390/jcm8020243

**Published:** 2019-02-13

**Authors:** Jong Chan Kim, Kang Su Cho, Do Kyung Kim, Doo Yong Chung, Hae Do Jung, Joo Yong Lee

**Affiliations:** 1 Department of Urology, Severance Hospital, Urological Science Institute, Yonsei University College of Medicine, Seoul 03722, Korea; LUMPAKCEF@yuhs.ac (J.C.K.); WJDENDYD1@yuhs.ac (D.Y.C.); 2 Department of Urology, Gangnam Severance Hospital, Urological Science Institute, Yonsei University College of Medicine, Seoul 06273, Korea; KSCHO99@yuhs.ac (K.S.C.); DOKYUNG@yuhs.ac (D.K.K.); 3 Department of Urology, Yongin Severance Hospital, Yonsei University Health System, Yongin 17046, Korea; HDJUNG@yuhs.ac

**Keywords:** urinary calculi, chemistry, uric acid, calcium oxalate

## Abstract

We analyzed the capacities of pertinent parameters (determined by single-energy non-contrast computed tomography [NCCT]) and urinary pH to predict uric acid stones. We reviewed the medical records of 501 patients whose stones were removed surgically or passed spontaneously between December 2014 and April 2016. Qualifying participants (*n* = 420) were stratified by the nature of the stone (calcium oxalate, uric acid, or infectious). Based on NCCT, we determined maximal stone length (MSL), mean stone density (MSD), and stone heterogeneity index (SHI) using Hounsfield units (HU) and calculated the variant coefficient of stone density (VCSD = SHI/MSD × 100). Urinary pH was also ascertained. Mean patient age was 55.55 ± 15.46 years. MSD (448.59 ± 173.21 HU), SHI (100.81 ± 77.37 HU), and VCSD (22.58 ± 10.55) proved to be significantly lower in uric acid versus other types of stones, as did urinary pH (5.33 ± 0.56; all *p* < 0.001). Receiver operating characteristic (ROC) curves depicting predictability of uric acid stones yielded area under ROC curve (AUC) values for MSD, SHI, VCSD, and urinary pH of 0.806 (95% CI: 0.761–0.850), 0.893 (95% CI: 0.855–0.931), 0.782 (95% CI: 0.726–0.839), and 0.797 (95% CI: 0.749–0.846), respectively, with corresponding cutpoints of 572.3 HU, 140.4 HU, 25.79, and 6.0. Among these four parameters, SHI was verifiably (DeLong’s test) the most effective predictor of uric acid stones (all *p* < 0.001). Compared with MSD, VCSD, and urinary pH, SHI may better predict uric acid stones, using a cutpoint of 140.4 HU.

## 1. Introduction

Urinary tract stones may be managed in a variety of ways. Medical treatment is one approach, including observation or urinary alkalization, and there are assorted non-medical alternatives, such as shock wave lithotripsy (SWL), percutaneous nephrolithotomy (PCNL), and surgical (ureteroscopic, laparoscopic, or open) stone extraction [1]. The location or size of a stone and its composition may help in deciding the proper modality and serve to predict the treatment outcome [2,3]. Uric acid stones fragment more readily by SWL or Holmium laser than do other stone types (e.g., calcium phosphate) [4,5,6] and seem particularly easy to treat. They are also amenable to medical management [7].

Various methods have been devised to predict stone composition. Although some trials have focused on defining stone composition through urinalysis, recent findings indicate that 24-hour urinalysis alone does not accurately predict the compositions of stones [8]. A recent report has shown no relationship between urinary pH and stone composition [9], possibly due to diurnal urinary pH fluctuations and resultant selection bias.

Given that non-contrast computed tomography (NCCT) has become the gold standard of diagnosing urinary stones [10,11], many groups have focused on the relation between Hounsfield units (HU), especially mean stone density (MSD), and urinary stone composition or treatment outcome [12]. In addition to MSD, the stone heterogeneity index (SHI), also known as standard deviation of HU, and the variant coefficient of stone density (VCSD) have emerged as potential therapeutic gauges [13,14]. The present study was thus conducted to assess the merit of MSD, SHI, VCSD, and urinary pH in predicting uric acid stones.

## 2. Materials and Methods

### 2.1. Patient Population

We retrospectively reviewed medical records of 501 patients who underwent surgical operations or procedures, or experienced spontaneous stone passage between December 2014 and April 2016 at our institution. Those lacking NCCT or stone composition data were excluded. Otherwise, the target stone assessed via NCCT and its composition were retrieved. Patients with bilateral ureteral calculi, urinary tract congenital anomalies, or single kidneys, and preoperative recipients of stone-dissolving medication (including potassium citrate, tiopronin, or antibiotics) were also disqualified. Surgical operations or procedures performed included ureteroscopic lithotripsy (URSL), SWL, PCNL, retrograde intra-renal surgery (RIRS), vesicolitholapaxy, laparoscopic ureterolithotomy, and laparoscopic pyelolithotomy. A total of 420 patients ultimately qualified for study. Our Institutional Review Board approved the study protocol (Approval No. 4-2018-0965). However, due to the retrospective design and the fact that all patient records/data were anonymized in advance, the requirement for written informed consent of subjects was waived.

### 2.2. NCCT Determinants and Stone Composition Analyses

Determinations based on NCCT included maximal stone length (MSL), MSD, SHI, and VCSD, using the GE Centricity system (GE Healthcare Bio-Sciences Corp, Piscataway, NJ, USA) for measuring purposes. MSL was defined as maximum stone diameter on the axial or coronal plane of NCCT. When magnifying the plane of NCCT for MSL, we assessed HU by setting the largest elliptical stone dimension as the region of interest. MSD was defined as the mean of HU in region of interest (ROI), SHI being the standard deviation of HU for the same the region of interest [13]. We calculated VCSD as SHI/MSD × 100 [14]. In addition to the above, pretreatment of urinary pH was also assessed. Quantitative analysis of stone composition was achieved by Fournier-transform infrared spectrometry, carried out at Green Gross Laboratories, Yongin, Korea.

### 2.3. Statistical Analyses

The entire patient cohort was stratified by stone composition, assigned to uric acid, calcium oxalate, or infectious groups. Data were expressed as mean ± standard deviation, except where otherwise indicated. Statistical comparisons of continuous patient demographic variables involved either Student’s or Welch’s two-sample *t*-test or the Wilcoxon rank-sum test. In subgroup analyses, one-way analysis of variance was used, thereafter comparing groups via the Tukey-Kramer post hoc test. To compare categorical variables, Pearson’s chi-squared test was invoked. We generated optimal cutpoints for significant values via receiver operating characteristic (ROC) curves, applying Youden methodology. All computations relied on R freeware v3.4.3 (R Foundation for Statistical Computing, Vienna, Austria; http://www.r-project.org), using the OptimalCutpoints package to determine optimal cutpoints, sensitivity, specificity, positive predictive value, and negative predictive value.

## 3. Results

**Figure 1:**
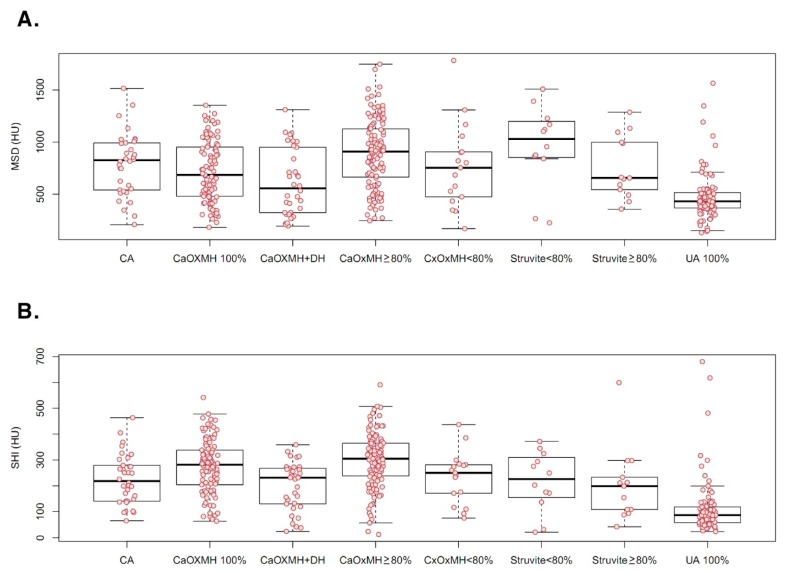
Mean stone density (MSD) (**A**) and stone heterogeneity index (SHI) (**B**) determined via non-contrast computed tomography and shown by stone composition. CA: carbonate apatite; CaOx: calcium oxalate; MH: monohydrate; DH: dihydrate; UA: uric acid; HU, Hounsfield unit.

**Figure 2:**
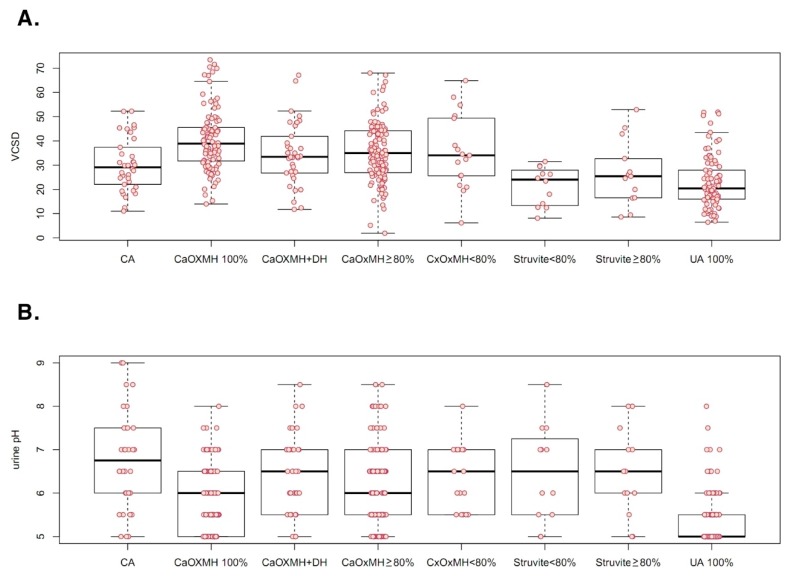
Variant coefficient of stone density (VCSD; determined via non-contrast computed tomography) (**A**) and urinary pH (**B**), shown by stone composition. CA: carbonate apatite; CaOx: calcium oxalate; MH: monohydrate; DH: dihydrate; UA: uric acid.

Mean patient age was 55.55 ± 15.46 years. The distribution of surgical operations and procedures was as follows: SWL, 11; laparoscopic pyelolithotomy, 3; laparoscopic ureterolithotomy, 30; PCNL, 63; RIRS, 106; URSL, 169; and vesicolitholapaxy, 25. There were 13 instances of spontaneous stone passage. Overall, 149 patients had renal stones, 248 had ureteral stones (upper ureter, 128; mid-ureter, 20; lower ureter, 100), and 23 had bladder stones. Mean values of determinants were as follows: MSL, 14.02 ± 10.90 mm; MSD, 722.49 ± 337.79 HU; SHI, 230.71 ± 125.22 HU; VCSD, 32.75 ± 13.72; and urinary pH, 6.05 ± 0.94 (Table 1). Stone analyses revealed calcium oxalate (CaOx) composition in 271, including 101 monohydrate stones, 119 mixed stones ≥80% CaOx monohydrate (MH), 17 mixed stones <80% CaOxMH, and 34 mixed CaOxMH and dihydrate (DH) stones. In the CaOx compound group of 271 patients, only one patient had a stone with 50% CaOxMH and 50% uric acid stone. In this group, only two patients had stones with 80% CaOxMH and 20% uric acid stone. Infectious calculi accounted for 34 carbonate apatite stones and 25 struvite stones. There were 90 instances of uric acid stones. Patient and stone characteristics are summarized according to composition groups in Table 2. As shown in Figure 1 and Figure 2, MSD (478.84 ± 232.41 HU), SHI (113.79 ± 106.79 HU), VCSD (22.93 ± 10.77), and urinary pH (5.41 ± 0.64) were significantly lower in instances of uric acid stones, compared with other stone types. In 420 patients, 27 took chemolytic agents including sodium bicarbonate and potassium citrate before treatment. However, in those, mean urine pH was 6.03 ± 0.81, and there was no difference comparing urine pH in total patients (*p* = 0.368).

Mean age, MSL, MSD, SHI, VCSD, and urinary pH differed significantly among the three composition groups: CaOx compounds (including MH and DH), infectious, and uric acid (Table 1). Based on post hoc analysis, patients with CaOx proved younger than those with uric acid stones (*p* = 0.001). MSL was also greater in uric acid stones, as opposed to those composed of CaOx compounds (*p* < 0.001); however, infectious stones surpassed uric acid stones in this regard (*p* = 0.043). Mean MSD of uric acid stones was significantly less than that of CaOx stones (*p* < 0.001) or infectious stones (*p* < 0.001). Mean SHI of uric acid stones also was significantly lower than that of CaOx stones (*p* < 0.001) or infectious stones (*p* < 0.001). In terms of SHI, infectious stones proved lower than CaOx compound stones (*p* = 0.002). Infectious and uric acid stones did not differ significantly in terms of VCSD (*p* = 0.060), but the VCSD of CaOx compound stones was significantly higher than both (all *p* < 0.001). Urinary pH in instances of uric acid stones was significantly lower than in instances of CaOx or infectious stones (all *p* < 0.001), and in instances of CaOx stones, urinary pH was significantly lower than in instances infectious stones (*p* < 0.001).

**Figure 3:**
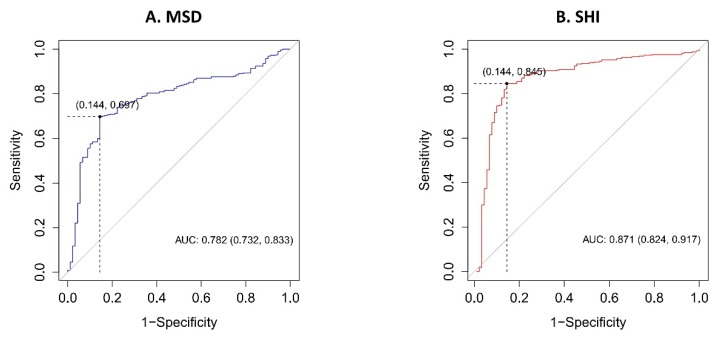
Receiver operating characteristic (ROC) curve of uric acid stones for (**A**) mean stone density (MSD), AUC = 0.782 (95% confidence interval [CI]: 0.732–0.833), cutpoint of 572.3 HU; (**B**) stone heterogeneity index (SHI), AUC = 0.871 (95% CI: 0.824–0.917), cutpoint of 140.4 HU. AUC, area under ROC curve; HU, Hounsfield unit.

**Figure 4:**
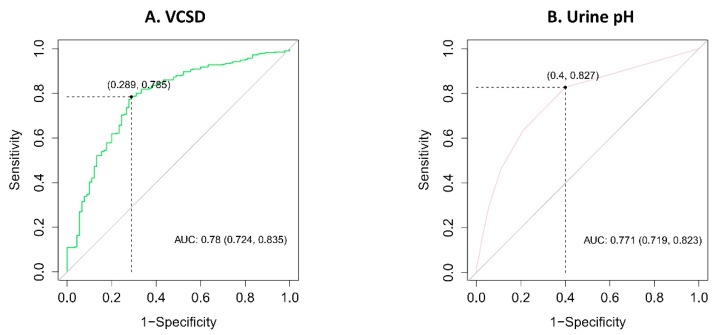
Receiver operating characteristic (ROC) curve of uric acid stones for (**A**) variant coefficient of stone density (VCSD), AUC = 0.780 (95% confidence interval [CI]: 0.724–0.835), cutpoint of 25.67; (**B**) urinary pH, AUC = 0.771 (95% CI: 0.719–0.823), cutpoint of 5.5. AUC, area under ROC curve.

For MSD, the area under the ROC curve (AUC) was 0.782 (95% confidence interval [CI]: 0.732–0.833), with a cutpoint of 572.3 HU, a sensitivity of 0.697 (95% CI: 0.644–0.746), a specificity of 0.855 (95% CI: 0.766–0.921), a positive predictive value of 0.946 (95% CI: 0.907–0.958), and a negative predictive value of 0.435 (95% CI: 0.377–0.602) in predicting uric acid stones (Figure 3A). The AUC of SHI was 0.871 (95% CI: 0.824–0.917), with a cutpoint of 140.4 HU, a sensitivity of 0.845 (95% CI: 0.802–0.883), a specificity of 0.856 (95% CI: 0.766–0.921), a positive predictive value of 0.955 (95% CI: 0.922–0.967), and a negative predictive value of 0.602 (95% CI: 0.528–0.748) in predicting uric acid stones (Figure 3B). The AUC of VCSD was 0.780 (95% CI: 0.724–0.835), with a cutpoint of 25.67, a sensitivity of 0.785 (95% CI: 0.767–0.828), a specificity of 0.711 (95% CI: 0.606–0.801), a positive predictive value of 0.908 (95% CI: 0.862–0.929), and a negative predictive value of 0.474 (95% CI: 0.0.408–0.597) in predicting uric acid stones (Figure 4A). The AUC of urinary pH was 0.771 (95% CI: 0.719–0.823), with a cutpoint of 5.5, a sensitivity of 0.827 (95% CI: 0.782–0.866), a specificity of 0.600 (95% CI: 0.491–0.702), a positive predictive value of 0.883 (95% CI: 0.830–0.911), and a negative predictive value of 0.486 (95% CI: 0.415–0.598) in predicting uric acid stones (Figure 4B). Using DeLong’s test for two correlated ROC curves, SHI surpassed MSD (*p* < 0.001), VCSD (*p* < 0.001), and urinary pH (*p* = 0.002) in the capacity to predict uric acid stones.

## 4. Discussion

Our results showed that SHI may better predict uric acid stones when using a cutpoint of 140.4 HU, compared to using MSD, VCSD, and urinary pH. In our data, SHI exceeded VCSD in predictability for the following reasons: (1) SHI of uric acid (versus all other) stones was very low by comparison (113.79 ± 106.79 HU versus 230.71 ± 125.22 HU), and (2) MSD showed a wider range in predicting the composition of various stones. MSD of all stones was 722.49 ± 337.79 HU, whereas MSD of uric acid stones was 478.84 ± 232.41 HU. In both instances, standard deviations were broader than that of SHI, perhaps diminishing its predictability.

Predicting the composition of urinary tract stones is an important aspect of their treatment. A major and fundamental reason is that composition and fragility are related. Cystine, calcium oxalate monohydrate, and brushite stones, for example, are denser and more resistant to SWL [3]. However, uric acid stones are also radiolucent, so despite their relative fragility, SWL is largely prohibitive [4,5]. The efficacy of Holmium laser is similarly a function of stone composition. Teichman et al. have reported that calcium oxalate monohydrate stones are the least prone to fragmentation when using a Holmium laser, whereas uric acid or cystine stones generally show moderate fragmentation [6]. Stone composition is a factor in treatment outcomes as well. Parks et al. have noted that patients with calcium phosphate stones are more refractory to SWL, requiring more treatments than patients with calcium oxalate stones [15]. Still another study has linked calcium phosphate stones to a lower rate of stone-free states after PCNL [16].

Separating uric acid stones from the rest is critical, because oral chemolysis (i.e., use of potassium citrate or sodium bicarbonate to alkalinize urine) is a viable therapeutic strategy [7]. Only ~50% of uric acid is in soluble form at a urinary pH of 5.75, but at a pH of 7.0, almost all uric acid in urine is in soluble form [17]. Uric acid is then apt to precipitate at low urinary pH, predisposing such patients to urate stones [18]. Unfortunately, recent studies have indicated a limited capacity to predict uric acid stones based on urinary pH [9]. In these studies, the main reason for the lack of a relationship between urine pH and stone composition may have been selection bias due to the diurnal variation of urine pH.

Aside from abnormal findings on urinalysis, an abundance of studies has been launched to define stone type via preoperative NCCT. Several have in fact found the MSD of uric acid (versus other) stones to be comparatively lower. Spettel et al. have confirmed a lower MSD for uric acid than for calcium stones [19], suggesting that an MSD of ≤500 in stones >4 mm is highly indicative of uric acid composition at urinary pH ≤5.5 (positive predictive value, 90%). Lee et al. have also recognized SHI as the standard deviation of stone density on NCCT [13], acknowledging its utility as an independent predictor of SWL success rates in patients with ureteral stones. Another relevant study has shown that SHI is helpful in predicting stone composition, especially in instances of uric acid stones in our previous study [20]. In this study, we analyzed 214 patients and found that SHI can be the most powerful factor in predicting uric acid stones before treatment. Finally, several recent reports have surfaced, addressing the use of dual-energy computed tomography (DECT) in differentiating stone type [21,22,23]. These studies underscore the efficacy of DECT in predicting stone composition, proving that this modality is particularly accurate in differentiating uric acid from non-uric acid stones. On the other hand, there are certain drawbacks to DECT, such as heightened radiation hazard (versus low-dose NCCT) and greater cost.

A number of studies addressing MSD and SHI have revealed correlations with treatment success rates, especially in terms of PCNL. Findings in most of these investigations seem to underscore that the higher the MSD value, the lower the treatment success rate [24,25,26]. Furthermore, a link between SHI and treatment outcomes is apparent. In our previous study, Lee et al. have recognized SHI as a prospective marker of stone fragility and confirmed its status as an independent predictor of SWL outcomes [13]. In addition, VCSD (calculated as SHI/MSD × 100) has recently emerged as a predictive index of treatment outcomes [27]. Yamashita et al. have reported an independent association between VCSD and SWL outcomes in patients with upper urinary tract calculi [14]. Therefore, we have attempted a new analysis of 501 patients to determine which factors, VCSD and SHI, predict uric acid stone. This is an entirely new study involving more than twice the number of subjects compared to our previous study [11].

The present study was a single-center retrospective effort, harboring a potential for selection bias. However, we enrolled a relatively large patient cohort with a variety of stone compositions, and RIRS and endoscopic-combined intrarenal surgery are now used more widely than in the past for treating urinary stones, enabling stone analysis in more patients [28,29]. Another issue is that the rate of uric acid stones in our study subjects exceeded the prevailing incidence of urate stones. This disparity likely reflects referrals of radiolucent uric acid stones (less treatable by SWL) from outlying facilities to our tertiary hospital. Uric acid stones can be associated with metabolic syndromes such as old age, chronic kidney disease, diabetes. Therefore, patients with uric acid stones are older, and the condition may develop more in men. Thus, uric acid stones can have a large stone size, low MSD, high SHI, and can be expected to develop in older men. In our study, we tried to find a way to accurately predict uric acid stones without 24-hour urine collection. Most importantly, uric acid stones are chemolysis-capable. Therefore, if we can predict that uric acid stones are present in pre-treatment patients before surgery, pretreatment chemolysis and surgery may increase stone-free rate. In our study, we attempted our analyses through the addition of VSCD, which is a new factor, and to increase the diagnostic predicting rate of uric acid stone by using several factors including MSD, VCSD, SHI, and urine pH before treatment. Especially, these factors can be helpful to predict uric acid stones before treatment and decide the treatment plan using chemolysis and chemoprevention using chemolytic agents, including sodium bicarbonate and potassium citrate. Although SHI is seemingly most useful in predicting uric acid stones, the other parameters studied proved predictive to lesser extents. In this particular setting, nomograms that fully incorporate the abovementioned parameters may offer the best predictive model.

## 5. Conclusions

Although MSD, VCSD, SHI, and urinary pH are all helpful in predicting uric acid stones, the predictability of SHI seemed best, using a cutpoint of 140.4 HU. Therefore, if MSD is less than 527 and SHI is less than 140, uric acid stones can be suspected. In addition, a urinary stone with urine pH of 5.5 or less and VCSD of 25.67 or less is more likely to be uric acid stone.

## Figures and Tables

**Table 1:** Summary of patient and urinary tract stone characteristics according to composition of calculi (calcium oxalate, infectious, and uric acid stones).

	Total	CaOx Compound	Infectious	Uric Acid	*p*-Value
No. of patients	420	271	59	90	
Age	55.55 ± 15.46	54.45 ± 15.75	52.51 ± 14.43	60.88 ± 14.07	0.001 ^a^
Sex					0.002 ^b^
Male	256 (60.95%)	164 (60.52%)	26 (44.07%)	66 (73.33%)	
Female	164 (39.05%)	107 (39.48%)	33 (55.93%)	24 (26.67%)	
Procedures					
SWL	11 (2.62%)	9 (3.32%)	1 (1.69%)	1 (1.11%)	
laparoscopic pyelolithotomy	3 (0.71%)	2 (0.74%)	0 (0.00%)	1 (1.11%)	
laparoscopic ureterolithotomy	30 (7.14%)	23 (8.49%)	5 (8.47%)	2 (2.22%)	
PCNL	63 (15.00%)	27 (9.96%)	19 (32.20%)	17 (18.89%)	
RIRS	106 (25.24%)	63 (23.25%)	10 (16.95%)	33 (36.67%)	
URSL	169 (40.24%)	133 (49.08%)	15 (25.42%)	21 (23.33%)	
vesicolitholapaxy	25 (5.95%)	5 (1.85%)	9 (15.25%)	11 (12.22%)	
Spontaneous passage	13 (3.10%)	9 (3.32%)	0 (0.00%)	4 (4.44%)	
Location					
Kidney	149 (35.48%)	77 (28.41%)	31 (52.54%)	41 (45.56%)	
Upper ureter	128 (30.48%)	93 (34.32%)	12 (20.34%)	23 (25.56%)	
Mid-ureter	20 (4.76%)	19 (7.01%)	0 (0.00%)	1 (1.11%)	
Lower ureter	100 (23.81%)	78 (28.78%)	8 (13.56%)	14 (15.56%)	
Bladder	23 (5.48%)	4 (1.48%)	8 (13.56%)	11 (12.22%)	
MSL	14.03 ± 10.90	11.05 ± 6.94	21.90 ± 16.96	17.84 ± 11.94	<0.001 ^a^
MSD	722.49 ± 337.79	782.60 ± 333.52	818.11 ± 324.46	478.84 ± 232.41	<0.001 ^a^
SHI	230.71 ± 125.22	272.10 ± 107.97	219.00 ± 110.24	113.79 ± 106.79	<0.001 ^a^
VCSD	32.75 ± 13.72	37.13 ± 12.99	27.63 ± 11.46	22.93 ± 10.77	<0.001 ^a^
Urine pH	6.05 ± 0.94	6.14 ± 0.87	6.61 ± 1.10	5.41 ± 0.64	<0.001 ^a^

Data expressed as mean ± standard deviation or number. CaOx: calcium oxalate; SWL: shock wave lithotripsy; PCNL: percutaneous nephrolithotomy; RIRS: retrograde intra-renal surgery; URSL: ureteroscopic lithotripsy; MSL: maximal stone length; MSD: mean stone density; SHI: stone heterogeneity index; VCSD: variant coefficient of stone density. ^a^ One-way ANOVA. ^b^ Pearson’s chi-squared tests with Yates’ continuity correction.

**Table 2:** Logistic regression models for predicting uric acid stones shown by test parameters.

	Odds Ratio	95% CI	*p*-Value
Univariate			
Age	1.032	1.015–1.050	<0.001
Sex (Male)	2.026	1.224–3.448	0.007
MSL	1.036	1.016–1.057	<0.001
MSD	0.996	0.995–0.997	<0.001
SHI	0.985	0.982–0.988	<0.001
VCSD	0.914	0.891–0.936	<0.001
Urinary pH	0.244	0.158–0.360	<0.001
Multivariate (with MSD & SHI)			
Age	1.033	1.010–1.057	0.005
Sex (Male)	1.516	0.766–3.048	0.236
MSL	1.052	1.021–1.087	0.001
MSD	0.998	0.996–1.000	0.038
SHI	0.989	0.984–0.994	<0.001
Urinary pH	0.359	0.226–0.543	<0.001
Multivariate (with VCSD)			
Age	1.021	1.001–1.042	0.048
Sex (Male)	1.859	1.012–3.495	0.049
MSL	0.997	0.971–1.022	0.791
VCSD	0.909	0.881–0.936	<0.001
Urinary pH	0.275	0.173–0.416	<0.001

MSL: maximal stone length, MSD; mean stone density, SHI; stone heterogeneity index, VCSD; variant coefficient of stone density.
